# Development and Validation of a Sensitive Liquid Chromatography-Tandem Mass Spectrometry (LC-MS/MS) Method for the Simultaneous Quantification of Thiopurine Nucleotides in Human Red Blood Cells

**DOI:** 10.7759/cureus.98953

**Published:** 2025-12-11

**Authors:** Maninder Singh, Sandeep Kaushal, Kanchan Gupta, Ajit Sood

**Affiliations:** 1 Pharmacology, Pandit Jawahar Lal Nehru Government Medical College and Hospital, Chamba, IND; 2 Pharmacology, Dayanand Medical College and Hospital, Ludhiana, IND; 3 Gastroenterology, Dayanand Medical College and Hospital, Ludhiana, IND

**Keywords:** 6-mercaptopurine, 6-thioguanine, lc-ms/ms, method validation, red blood cells, therapeutic drug monitoring, thiopurine metabolites

## Abstract

Background

Therapeutic drug monitoring (TDM) of thiopurine metabolites in red blood cells (RBCs) is essential to optimizing dosing regimens and minimizing adverse effects. Current analytical methods lack the desirable specificity and sensitivity needed for optimum quantification at therapeutic concentrations on a routine basis. The objectives of the current study were to validate and develop a highly repeatable and specific liquid chromatography-tandem mass spectrometry (LC-MS/MS) method for the simultaneous quantification of 6-mercaptopurine (6-MP), 6-thioguanine (6-TG), and 6-methylmercaptopurine (6-MMP) within human red cells.

Methods

Hydrophilic interaction liquid chromatography (HILIC) was employed for separation using a gradient elution system. Ammonium formate with formic acid in water (A) and acetonitrile (B) was used as the mobile phases. Multiple reaction monitoring (MRM) detection was carried out in positive mode using an electrospray ionization (ESI) source. The method was validated according to International Council for Harmonisation of Technical Requirements for Pharmaceuticals for Human Use (ICH) guidelines and included testing for linearity, accuracy, recovery, precision, and specificity in the 0.5-1000 ng/mL concentration range.

Results

The developed method showed excellent linearity for the three analytes with a coefficient of determination (R²) of ≥0.9997. Recovery analysis showed acceptable accuracy, with the mean recoveries between 88.74% and 117.37% for all the concentration levels. The method showed high specificity without interference from endogenous matrix components. Precision study showed a relative standard deviation (RSD) of ≤15% for all the analytes.

Conclusions

The developed LC-MS/MS approach gives a reliable technique for the simultaneous quantification of thiopurine metabolites within erythrocytes. With its reliability and sensitivity, it makes a suitable approach for therapeutic drug monitoring at the clinical level, therefore contributing toward enhanced thiopurine therapy under the banner of personalized medicine.

## Introduction

Thiopurines, such as azathioprine (AZA) and 6-mercaptopurine (6-MP), and their active metabolites are a cornerstone of treatment for various clinical disorders under an immunosuppressant drug regimen. They are extensively used for treating inflammatory bowel disease (IBD), acute lymphoblastic leukemia (ALL), and many autoimmune disorders such as systemic lupus erythematosus and rheumatoid arthritis [1]. Their therapeutic activities are nearly entirely dependent on their active metabolites, notably 6-thioguanine (6-TG) nucleotide and 6-methylmercaptopurine (6-MMP), which manifest their immunosuppressant activities by their incorporation into DNA and purine synthesis inhibitor activities [2].

It is, however, challenging to employ thiopurines for clinical therapeutics due to their application being marred by immense interindividual variation between patients in their drug exposure and therapeutic outcome. Such variation occurs primarily based on genetic variation between patients in their major metabolic enzymes, thiopurine S-methyltransferase (TPMT), inosine triphosphate pyrophosphatase (ITPA), and nudix hydrolase 15 (NUDT15) [3]. Eventually, patients end up having therapeutic failure due to suboptimal drug levels arising due to inadequate drug exposure and serious adverse effects, which manifest as serious, life-endangering myelosuppression and hepatotoxicity [4].

Therapeutic drug monitoring (TDM) of thiopurine metabolites within red blood cells (RBCs) has been proposed to be a very valuable clinical tool, allowing for the fine-tuning of therapy while reducing side effects [5]. RBCs offer a perfect matrix for measuring thiopurine nucleotide concentrations, as they cannot synthesize purines de novo, making them highly responsive to thiopurine incorporation. In addition, their long lifetime, that is, 120 days, provides a measure of cumulative drug exposure [6]. Existing analytical techniques used for thiopurine metabolite quantification are high-performance liquid chromatography (HPLC) and liquid chromatography-tandem mass spectrometry (LC-MS/MS) [7]. While used extensively, HPLC is, however, often deficient in terms of sensitivity and specificity needed for the accurate quantification at therapeutic doses. LC-MS/MS techniques are more sensitive and selective, but they need careful optimization and validation to ensure they are effective in clinical use. This work sought to develop and validate a robust, highly sensitive LC-MS/MS protocol for the simultaneous determination of 6-MP, 6-thioguanine (6-TG), and 6-methylmercaptopurine (6-MMP) from human red cells [8]. The validated method hopes to provide a reliable analytical tool for therapeutic drug monitoring, thus enabling personalized medicine approaches in thiopurine therapy optimization.

## Materials and methods

Reagents and chemicals

Reference standards of 6-MP (Chemical Abstracts Service {CAS}: 6112-76-1), 6-TG (CAS: 154-42-7), and 6-MMP (CAS: 33312-93-5), along with dithiothreitol (DTT), were procured from Sigma-Aldrich (USA). LC-MS-grade acetonitrile and ultrapure water were supplied by Honeywell International Inc. Ammonium formate and formic acid of analytical grade were also procured from Sigma-Aldrich. All chemicals and reagents were stored according to manufacturer recommendations and used within their specified shelf life.

Instrumentation

All the analytical procedures were conducted using an Agilent 1260 Infinity (Agilent Technologies, Santa Clara, CA) LC-MS/MS system, equipped with a binary pump, an autoinjector featuring temperature control, and a thermostatic column compartment, all connected to a triple quadrupole mass spectrometer. A HILIC column (2.1 × 150 mm, 1.8 μm) was employed to achieve chromatographic separation. Additional equipment included an analytical balance with 0.00001 g readability, an ultrasonic bath, a vortex mixer, a centrifuge capable of 7000 revolutions per minute (rpm), and 0.22 μm polyvinylidene difluoride (PVDF) syringe filters.

Data processing and statistical analysis

Chromatographic peak integration and quantification were performed using Agilent MassHunter Quantitative Analysis software (Agilent Technologies, Santa Clara, CA). Calibration curves were constructed using weighted (1/x) linear regression, and the coefficient of determination (R²) was automatically calculated by the software. Accuracy (%) was calculated as follows: accuracy = (measured concentration​/nominal concentration) × 100.

Precision was reported as relative standard deviation (RSD%): RSD = (SD/mean​) × 100. For intra-assay precision, six replicates (n = 6) were analyzed at each Quality control (QC) level within a single analytical run.

Chromatographic and MS conditions

The mobile phase consisted of 5 mM ammonium formate with 0.1% formic acid in water (phase A) and acetonitrile (phase B). Chromatographic separation was performed under isocratic conditions at a flow rate of 0.3 mL/minute. The column was maintained at 40°C, with an injection volume of 10 μL, and the autosampler temperature was controlled between 2°C and 8°C. The total analytical run time was 20 minutes, followed by a two-minute equilibration period. Detection was carried out using positive electrospray ionization (ESI) in multiple reaction monitoring (MRM) mode, with ion source parameters optimized to maximize analyte sensitivity while ensuring specificity and reproducibility.

Standard solution preparation

Stock solutions were prepared at concentrations of 1000 μg/mL for 6-TG and 100 μg/mL for 6-MP and 6-MMP in methanol and stored at -20°C. Serial dilutions are used to obtain working solutions. Calibration curves were constructed across 0.5-1000 ng/mL using six concentration levels

Collection and processing of samples

Whole blood samples were withdrawn and collected in Ethylenediaminetetraacetic acid (EDTA) tubes from healthy volunteers after informed consent and ethical approval. The Research and Ethics Committee of Dayanand Medical College and Hospital issued approval DMCH/P/2018. Samples were centrifuged to separate plasma and buffy coat, which were subsequently discarded. Red blood cells were washed three times with normal saline and stored at -80°C until analysis. For method validation studies, red blood cell samples were spiked with known concentrations of 6-TG, 6-MP, and 6-MMP standards. Samples underwent filtration through 0.22 μm PVDF filters before injection into the LC-MS/MS system.

Validation

The specificity of the analytical method was evaluated by examining blank matrices, individual standard solutions, and mixed standard solutions to ensure the absence of interfering peaks at the retention times corresponding to the target analytes. Linearity was assessed over a broad concentration range of 0.5-1000 ng/mL for each analyte, employing six calibration points. Linear regression analysis was performed, and the coefficient of determination (R²) was calculated to confirm the proportional relationship between concentration and detector response. Accuracy and precision were established through recovery studies conducted at four concentration levels, 0.5, 10, 100, and 1000 ng/mL, with duplicate analyses performed at each level. Percent recovery and relative standard deviation (RSD%) were computed to evaluate the method’s reliability and reproducibility across the tested concentration range.

## Results

Method specificity

**Figure 1:**
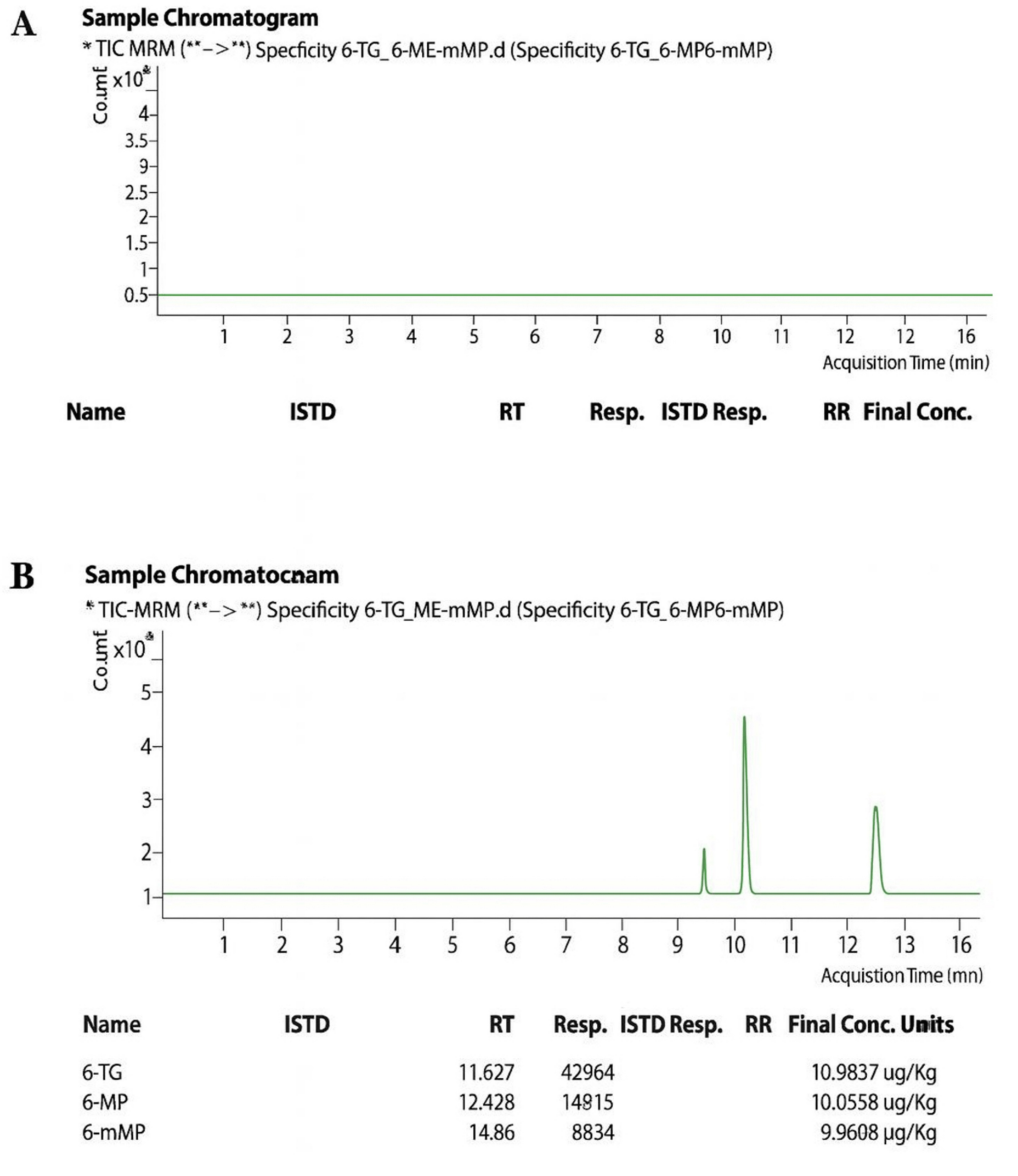
Monitoring chromatogram (A) Blank blood sample. (B) Blood sample spiked with 6-TG, 6-MP, and 6-MMP *Primary MRM transition used for analyte identification/confirmation **Secondry MRM transition used for analyte identification/confirmation 6-MP, 6-mercaptopurine; 6-TG, 6-thioguanine; 6-MMP, 6-methylmercaptopurine; TIC, total ion chromatogram; MRM, multiple reaction monitoring; ISTD, internal standard; RT, retention time; RR, relative response

Specificity studies demonstrated the method’s ability to accurately identify and quantify desired analytes without interference from endogenous matrix components. For this, the rational analysis of blank red blood cell matrices was carried out and showed no peaks at the retention times corresponding to 6-MP, 6-TG, or 6-MMP. Individual standard solutions produced distinct, well-resolved peaks with appropriate retention times and mass spectral characteristics (Figure 1).

Linearity

The calibration curve was generated by analyzing 6-MP, 6-TG, and 6-MMP standards spiked into blank blood and plotting their peak area ratios. Linearity was evaluated using linear regression analysis, which demonstrated excellent correlation for all three analytes across the tested concentration spectrum (0.5-1000 ng/mL). The following linear relationships were established: 6-TG: $y = 3934.9x - 251.85(R^{2} =0.9999)$; 6-MP: $y = 1476.9x - 40.25(R^{2} =0.9997)$; and 6-MMP: $y = 890.72x - 43.92(R^{2} =1.0000)$.

**Figure 2:**
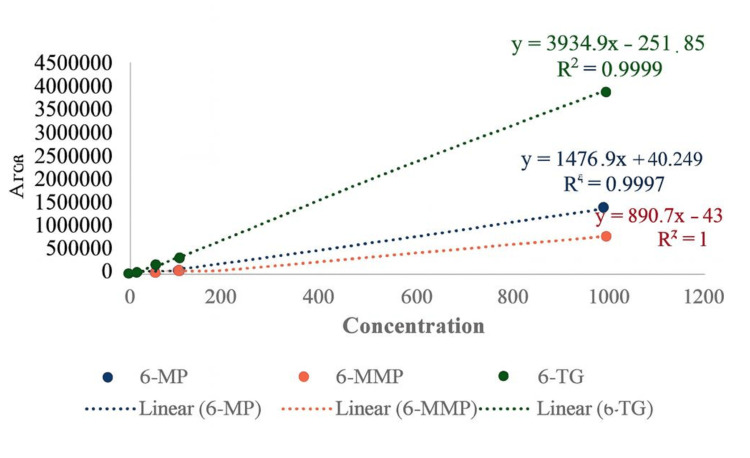
Linearity curve of 6-TG, 6-MP, and 6-MMP 6-MP, 6-mercaptopurine; 6-TG, 6-thioguanine; 6-MMP, 6-methylmercaptopurine

All correlation coefficients exceeded 0.99 or better, indicating excellent linearity and method reliability across the analytical range. The lower limit of quantification (LLOQ) was defined as the lowest point on the calibration curve that could be quantified with acceptable accuracy and precision. At this level, the analyte response had to be a minimum of five times higher than the blank response. For each standard concentration, the acceptance criterion was set at a deviation of ±15% from the nominal value, except at the LLOQ, where a deviation of up to 20% was considered acceptable (Figure 2).

Accuracy and recovery

Intra-assay precision and accuracy were assessed using six replicates of 6-TG, 6-MP, and 6-MMP at four quality control (QC) concentrations: 0.5 ng/mL (LLOQ), 10.0 ng/mL (lower quality control {LQC}), 100 ng/mL (middle quality control {MQC}), and 1000 ng/mL (higher quality control {HQC}) (Table 1). The assessment of inter-assay precision was performed by testing the same four QC concentrations in four independent analytical runs. Data were considered acceptable when accuracy was within 85%-117% of the nominal values and precision did not exceed ±15% relative standard deviation (RSD). For the LLOQ, the acceptance limits were broader, requiring accuracy within 80%-120% and precision not exceeding 20%. Recovery studies demonstrated acceptable accuracy across all tested concentration levels. Mean recovery percentages ranged from 88.74% to 117.37%, well within acceptable limits for bioanalytical methods (typically 85%-115%) (Table 2).

**Table 1:** Percentage recovery of 6-TG, 6-MP, and 6-MMP at four quality control (QC) concentrations 6-MP, 6-mercaptopurine; 6-TG, 6-thioguanine; 6-MMP, 6-methylmercaptopurine

Concentration (ng/mL)	6-TG Recovery (%)	6-MP Recovery (%)	6-MMP Recovery (%)
0.5	106.02-116.08	102.34-111.96	107.54-111.13
10.0	102.96-115.87	111.04-113.81	106.60-117.37
100.0	88.74-98.37	91.14-94.08	98.08-98.99
1000.0	99.62-101.92	99.09-114.22	99.53-101.23

**Table 2:** Accuracy, precision, and reproducibility of 6-TG, 6-MP, and 6-MMP at four quality control concentrations 6-MP, 6-mercaptopurine; 6-TG, 6-thioguanine; 6-MMP, 6-methylmercaptopurine; LQC, lower quality control; MQC, middle quality control; HQC, higher quality control; LLOQ, lower limit of quantification

	Reference Concentration	Mean ± SD (ng/mL)	Reproducibility	Accuracy, %
LLOQ	0.5 ng/mL	0.546 ± 0.019	3.50	109.18%
LQC	10 ng/mL	10.961 ± 0.611	5.57	111.32%
MQC	100 ng/mL	94.90 ± 4.26	4.49	95.02%
HQC	1000 ng/mL	1012.52 ± 57.53	5.68	103.94%

Method performance

The method demonstrated strong validation characteristics, including superior sensitivity, specificity, and reproducibility, with the lower limit of quantification (LLOQ) defined at 0.5 ng/mL for all analytes, providing adequate sensitivity for therapeutic drug monitoring applications.

## Discussion

The present study aimed to develop and validate a sensitive and reproducible LC-MS/MS assay for the simultaneous quantification of 6-TG, 6-MP, and 6-MMP in human RBCs. Measuring thiopurine metabolites is important to guide the appropriate management of therapy in patients with IBD, ALL, and autoimmune disease, which includes a goal and individualized dosing [3,9].

By employing HILIC with a triple quadrupole detector, our method achieved high analytical efficiency, with linearity, precision, and sensitivity, meeting ICH Q2(R1) validation specifications [6]. Specificity analysis confirmed the absence of interfering peaks during the retention times of blank RBC matrices for analytes, giving a clean baseline and sharp identification of target analytes. Clear chromatographic separations of 6-TG, 6-MP, and 6-MMP were observed, enabling precise quantitative analysis without cross-interference. This is similar to observations from previous LC-MS/MS-based research, which depicted that tandem mass spectrometry provides improved selectivity and low matrix effects compared to HPLC-UV systems [10-12]. As compared to a previous study by Dervieux and Boulieu (1998), where overlap between thiopurine metabolites limited sensitivity and reproducibility, the new method overcomes it through the employment of optimized positive MRM mode electrospray ionization for the concurrent identification of all three analytes in a single run [13].

Calibration curves for all three analytes were highly linear in the concentration range of 0.5-1000 ng/mL (R² > 0.9997). The slopes of the calibration equations (6-TG: 3934.9x - 251.85; 6-MP: 1476.9x + 40.25; 6-MMP: 890.72x - 43.92) demonstrated an excellent detector response that was proportional to analyte concentration. This is comparable to previous LC-MS/MS assays that reported either similar or improved linearity performance (e.g., Bajaj et al. (2022) [10] reported R² > 0.999 for 6-TG and 6-MMP over the 0.2-7.5 μmol/L and 4-150 μmol/L, respectively, while Hofmann et al. (2012) [11] found linearity up to 1 nmol/injection for thiopurine nucleotides to R² > 0.998). In this study, the LLOQ for each analyte was 0.5 ng/mL, which is much more sensitive than the 0.1 μmol/L and 0.15 μmol/L LLOQs for 6-TG that were reported by Mei et al. (2017) [12] and Yu et al. (2023) [14], respectively. In molar equivalence, 0.5 ng/mL represents several times greater sensitivity and can quantify concentrations that are not therapeutic but can be used in early evaluations of pharmacokinetics and patients on low doses. This level of sensitivity will enable the detection of underexposure to thiopurines in patients with low TPMT or NUDT15 activity [3]. The extended upper limit of quantification (ULOQ) of 1000 ng/mL ensures the coverage of a wide therapeutic window, thereby enhancing the method’s applicability for both clinical and pharmacological studies.

Precision and accuracy adhered to acceptance limits outlined by ICH Q2(R1), with intra- and inter-assay RSD below 15% and accuracy between 85% and 117%. Mean recovery figures ranged from 88.74% to 117.37% for all quality controls, corresponding to acceptable intervals for bioanalytical assays (85%-115%). Relative to other validated protocols, the current work reveals equivalent or improved recovery efficiency relative to other validated protocols. Dervieux and Boulieu (1998) achieved recoveries of 73.1% for 6-TG and 84.0% for 6-MMP for red blood cells, reflecting limitations of rudimentary HPLC-based protocols [13]. Hofmann et al. (2012) reported accuracy between 92% and 107% for thiopurine nucleotide standards by LC-MS/MS, a similar result here [11]. Such results confirm a successful sample preparation protocol, including proper washings, deproteinization, and filtration, for extracting intracellular metabolites efficiently without degradation. Optimized HILIC conditions favored reproducible analyte retention and suppressed ion suppression [15,16]. Such analytical thoroughness agrees with newer LC-MS/MS-based validation reports, for example, Wróblewski et al.’s (2023) report for a cytisine quantification assay, which similarly emphasized matrix purity and stable extraction recovery as determinants of high reproducibility [17].

Comparisons across leading methods in the literature, as indicated in Table 3, highlight that the current assay achieved one of the lowest reported lower limit of quantification (LLOQ) for thiopurines (0.5 ng/mL), with Mei et al. (2017) [12] and Bajaj et al. (2022) [10] reporting a higher lower limit, and Hofmann et al. (2012) [11] required a much larger injection volume to achieve an equivalent sensitivity [14]. Additionally, recovery values in this assay surpassed those reported by Dervieux and Boulieu (1998), and the sensitivities observed are a result of extraction chemistry advancements, along with improved mass spectrometric efficiency based on higher resolving power [13]. The LLOQ to ULOQ of 0.5-1000 ng/mL is inclusive of levels encountered therapeutically and supra-therapeutically in patient samples. This is important, and it facilitates pharmacokinetic analysis and therapeutic drug monitoring (TDM). The importance of comprehensive thiopurine quantification has been thoroughly demonstrated in the context of various diseases, with baseline measurements of 6-TG and 6-MMP assisting clinicians in balancing efficacy and toxicity [2,18]. Levels of 6-TG above 230-260 pmol/8 × 10⁸ RBC are correlated with disease remission in patients suffering from inflammatory bowel disease, while excessive levels of 6-MMP can be associated with hepatotoxicity [9,19]. It is noted that the sensitivity and linearity demonstrated in the assay indicate that the assay developed can be utilized in the purpose of therapeutic monitoring, particularly as patient variability may exist based on TPMT or NUDT15 polymorphisms, which may necessitate alteration to the dosing of thiopurines [3,20]. The use of LC-MS/MS further enhances precision and allows for differentiation between active and methylated metabolites, unlike UV-based detection methods, which often struggle with overlapping peaks and higher noise levels [14].

**Table 3:** Comparison of analytical methods for thiopurine metabolite quantification 6-MP, 6-mercaptopurine; 6-TG, 6-thioguanine; 6-MMP, 6-methylmercaptopurine; LLOQ, lower limit of quantification; ULOQ, upper limit of quantification; LC-MS/MS, liquid chromatography-tandem mass spectrometry; HPLC, high-performance liquid chromatography; N/A, not available; TGN, thioguanine nucleotide; QCs, quality controls; ICH, International Council for Harmonisation of Technical Requirements for Pharmaceuticals for Human Use; TGMP, thioguanosine monophosphate; TGDP, thioguanosine diphosphate; TGTP, thioguanosine triphosphate; MeTIMP, methyl-thioinosine monophosphate

Method (Author and Year)	Technique	Analytes Measured			LLOQ		ULOQ		Recovery (%)
		6-TG	6-MMP	6-MP	6-TG	6-MMP	6-TG	6-MMP	
Current study	LC-MS/MS	6-TG	6-MMP	6-MP	0.5 ng/mL	0.5 ng/mL	1000 ng/mL	1000 ng/mL	88.74-117.37 (mean across levels)
Mei et al. (2017) [12]	LC-MS/MS	6-TG	6-MMP	N/A	0.1 μmol/L	0.1 μmol/L	N/A	N/A	N/A
Bajaj et al. (2022) [10]	LC-MS/MS	6-TG	6-MMP	N/A	0.2 μmol/L	4 μmol/L	7.5 μmol/L	150 μmol/L	N/A
Yu et al. (2023) [14]	HPLC-UV	6-TG	6-MMP	N/A	0.15 μmol/L	1 μmol/L	15 μmol/L	100 μmol/L	N/A (validated per FDA/ICH; acceptable limits met)
Hofmann et al. (2012) [11]	LC-MS/MS	Multiple nucleotides (e.g., TGMP, TGDP, TGTP, and MeTIMP; summed as TGN equivalents)	-	N/A	~0.5 pmol/injection (most analytes)	-	N/A	-	92-107 (accuracy in spiked QCs)

Validation herein was ample but conducted using spiked samples in a healthy red blood cell matrix and not patient samples. Thus, a full assessment of any interferences occurring due to pathological matrices, concomitant medications, or metabolite interferences​​​ could not be carried out. Future research should entail clinical validations across patient populations while attempting to correlate concentrations measured with pharmacogenetic and clinical outcomes. Another limitation was no testing for metabolite stability under different storage conditions, a factor necessary when standardizing sample handling in multicentric trials.

## Conclusions

In conclusion, the developed LC-MS/MS assay offers a highly sensitive, accurate, and specific approach for simultaneously quantifying 6-TG, 6-MP, and 6-MMP in red blood cells. With an LLOQ of 0.5 ng/mL, excellent linearity (R² > 0.9997), and recovery values between 88.74% and 117.37%, its analytical performance matches or exceeds previously reported methods and fully encompasses the therapeutic range required for clinical thiopurine monitoring. This platform holds potential for applications in pharmacogenomic-guided dosing, longitudinal therapeutic drug monitoring, and high-throughput workflows in personalized medicine. When integrated with TPMT and NUDT15 genotyping, it may further enhance the prediction of treatment outcomes. However, the present findings represent only preliminary validation, and additional stability studies are needed before routine clinical implementation. While LC-MS/MS provides clear analytical advantages, its high setup and instrumentation costs may limit wider adoption, whereas HPLC remains more accessible but lacks the sensitivity required for precise therapeutic drug monitoring.
